# A Prospective Hospital-Based Study on the Clinico-Etiological Profile of the First Episode of a Seizure in Children

**DOI:** 10.7759/cureus.31242

**Published:** 2022-11-08

**Authors:** Prashanthi M, Sai Chandar Dudipala, Roop Shankar, Raja V Reddy, Amith Kumar Ch

**Affiliations:** 1 Pediatrics, Prathima Institute of Medical Sciences, Karimnagar, IND; 2 Pediatric Neurology, Star Women & Children Hospital, Karimnagar, IND; 3 Paediatrics, Star Women & Children Hospital, Karimnagar, IND; 4 Pediatrics, Star Women & Children Hospital, Karimnagar, IND

**Keywords:** electroencephalogram, epilepsy syndromes, febrile seizures, etiology, international league against epilepsy (ilae), first seizures

## Abstract

Background

This study aims to examine the clinico-etiological profile of children with the first episode of a seizure and categorize seizure types based on age groups in a tertiary care hospital.

Methodology

This was a prospective observational study conducted at a tertiary care medical institute in India over two years. Children (one month to 12 years of age) with the first episode of a seizure were included in the study population. The data collected included demographic profile, history, examination, biochemical profile, electroencephalogram (EEG), and neuroimaging. Children were categorized as generalized-onset, focal-onset, and unknown-onset seizures based on the International League Against Epilepsy 2017 seizure classification. Children were also classified according to specific etiologies such as structural, metabolic, or other specific causes. All the children were followed up at the hospital's outpatient clinic or through a telephonic interview.

Results

A total of 220 children were examined in this study. Among them, 64% were male and 36% were female, with a male-to-female ratio of 1.75:1. Among the 220 children, 21.8% had a family history of seizure. The most common type of seizures were generalized-onset seizures (n = 110, 50%), followed by focal-onset seizures (n = 96, 43.6%). Overall, 9% of children presented with status epilepticus as their first-episode seizure. An abnormal EEG was recorded for 122 (76%) children. While 60% of children had abnormal neuroimaging findings, the remaining had normal neuroimaging. Febrile seizures (n = 92, 41.9%) were the most common cause of the first episode of a seizure. Most of the febrile seizures occurred between the age of one and four (n = 60, 65.2%). Epilepsy syndromes were the second most common etiology, followed by central nervous system (CNS) infections, structural brain abnormalities, metabolic disorders, vascular lesions, toxins, and immune-mediated causes, in that order. In 14 (6.36%) children, the etiology was unknown at the time of the study.

Conclusions

First-episode seizures in children cause physical, mental, and financial stress on the parents. The collection of detailed history, examinations, and appropriate investigations can help identify the etiology of seizures. It was possible to determine the etiology of the first episode of a seizure in the majority of the patients. Generalized-onset seizures were the most common. Febrile seizures, epilepsy syndrome, CNS infections, vascular lesions, and metabolic disorders were the main etiological factors, in that order.

## Introduction

Seizures are one of the most common presenting complaints in pediatric practice. A seizure is defined as “a transient occurrence of signs and/or symptoms due to abnormal excessive or synchronous neuronal activity in the brain” [1]. Approximately 10% of the population will experience seizures in their lifetime [2,3]. Half of them will experience seizures before the age of 18, and the highest seizure occurrence is before the age of one [4].

The first seizure episode can have tremendous mental and physical consequences for the child and the family. Hence, the physician is required to conduct a thorough evaluation of the cause of the seizure and proper management to alleviate the stress on the child and the family.

Compared to older children, infants show different characteristics of seizures [5]. The etiological causes also vary in early seizures, including metabolic issues, infection, and structural anomalies of the brain.

Hence, the correct assessment and scrutiny of the first episode of a seizure is very important to diagnose the index event as epilepsy and further evaluate the recurrence risk. This will also aid in the decision-making of the initiation of antiseizure medications for the child.

Seizure characteristics have been well described and classified for the adult population. However, there is limited research on the profile of seizures in younger children. This study is conducted to contribute more data to the literature on the clinico-etiological profile of the first episode a seizure in children.

This study aims to examine the clinico-etiological profile of the first episode of a seizure in children and categorize seizure types based on children's age group in a tertiary care hospital in India.

## Materials and methods

This prospective observational study was conducted for two years, from November 2018 to October 2020, in a tertiary care hospital in India. The study was approved by the ethical committee of the institution.

Study population

The study population was selected according to the inclusion and exclusion criteria. Children between the age of one month and 12 years who were admitted to the hospital or visited the outpatient clinic of the hospital with the first episode of a seizure were included in the study. Neonates and children who were older than 12 and had experienced more than one episode of seizure were excluded. Informed written consent was procured from the participants’ parents or guardians. Data were collected using a pre-designed structured pro forma.

In the pro forma, the parents or guardians provided details on the participants, including age, gender, socioeconomic status, consanguinity of parents, seizure semiology, history of prior provoked seizure, history of neurological insult, developmental history, birth details, and family history of seizure or epilepsy. All the children participants underwent a detailed physical and neurological examination, and the findings were recorded.

Laboratory investigations, including blood glucose, serum electrolytes, and calcium, were performed as necessary. Magnetic resonance imaging (MRI) of the brain was carried out when clinically indicated. An electroencephalogram (EEG) was also performed when indicated.

The children were categorized based on the latest International League Against Epilepsy (ILAE) 2017 seizure classification as generalized onset, focal onset, and unknown onset [6]. Further, they were classified based on specific etiologies such as structural, metabolic, or other causes. Some children were initiated with antiseizure medications appropriately. After enrollment, all the participants were followed up at the hospital’s outpatient clinic or through a telephonic interview. Data were statistically analyzed using the SPSS software (IBM Corp., Armonk, NY, USA).

## Results

In this study, a total of 220 children were included. Among them, 140 (64%) were male and 80 (36%) were female, resulting in a male-to-female ratio of 1.75:1. In the sample, 68 (31.9%) had consanguineous parents. Only 16 (7.27%) children had a low birth weight (<2.5 kg). In total, 28 (12.7%) were admitted to the neonatal intensive care unit (NICU) for various reasons. Overall, 18 (8.2%) children had developmental delays and 48 (21.8%) children had a family history of seizures (Table 1).

**Table 1:** Demographic details. NICU - neonatal intensive care unit

Criterion	Number of children	Percentage (%)
Age distribution		
1 month – 1 year	46	20.9
1–4 years	102	46.36
5–8 years	50	22.7
9–12 years	22	10
Sex distribution		
Male	140	64
Female	80	36
Special characteristics		
Consanguinity	68	32
Low birth weight	16	7.27
NICU stay	28	12.7
Developmental delay	18	8.2
Family history of seizures	48	21.8

Seizures were classified according to the ILAE 2017 classification as generalized onset, focal onset, unknown onset, and focal to bilateral tonic-clonic seizures (Table 2) [6]. Half of the children had generalized-onset seizures. Among children with generalized-onset seizures, 56 (51%) presented with tonic seizures and 48 (43.6%) with tonic-clonic seizures. Among those with focal-onset seizures (96), 42 (43.75%) had the clonic type and 34 (35.4%) had the tonic type. In total, 20 (9.0%) children presented with status epilepticus in their first-episode seizure.

**Table 2:** Seizures type according to ILAE 2017 [6]. ILAE - International League Against Epilepsy

Seizure type	Number of children	Percentage (%)
A. Generalized onset	110	50
Tonic-clonic	48	43.6
Clonic	2	1.8
Tonic	56	50.9
Epileptic spasms	2	1.8
Typical absence	2	1.8
B. Focal onset	96	43.6
Clonic	42	43.75
Tonic	34	35.4
Automatisms	2	2.08
Autonomic	4	4.16
Behavioral arrest	14	14.58
C. Unknown onset	6	2.7
D. Focal to bilateral clonic	6	2.7
Status epilepticus	20	9.0

In this study, an EEG was performed on 160 (72.72%) children. Among them, 38 (23.75%) children had a normal record, 38 (23.75%) had generalized slowing, 18 (11.2%) had temporoparietal spikes, and 16 (10%) had focal slowing with spikes. Generalized epileptiform abnormalities, focal slowing, and centrotemporal spikes were noticed in 14 (8.7%), 12 (7.5%), and 10 (6.2%) children, respectively. The remaining children had temporal spikes (six children), occipital spikes (four children), and other non-specific abnormalities (four children). Neuroimaging was done in 132 (60%) children, among which 68 (51.5%) had normal imaging. The remaining children had various abnormal findings (Table 3).

**Table 3:** Neuroimaging findings.

Finding	Number of children	Percentage (%)
Total neuroimaging	132	60
Normal	68	51.5
Abnormal	64	48.5
Meningeal enhancement	12	9.1
Neurocysticercosis	10	7.6
Focal cortical dysplasia	6	4.5
Edema	6	4.5
Gliotic lesion	8	6.0
Vascular lesions	10	7.6
White matter changes	4	3.0
Other findings	8	6.0

Etiological distribution was conducted according to the 2017 ILAE epilepsy framework. Febrile seizures were considered separate entities. The most common etiology for first-episode seizures in children was febrile seizures (92; 41.9%). Epilepsy syndromes, CNS infections, metabolic causes, vascular etiology, toxins, and immunological diseases were observed as the etiology in 28 (12.7%), 26 (11.8%), 14 (6.4%), 10 (4.5%), 8 (3.6%), and 6 (2.7%) children, respectively. In 14 (6.4%) children, the etiology was unidentified (Table 4). Age-wise distribution of etiology was also carried out.

**Table 4:** Etiological distribution.

Etiology	Number of children	Percentage (%)	
Febrile seizures	92	41.9	
Infections	26	11.8	
Structural	22	10	
Metabolic	14	6.4	
Immune	6	2.7	
Vascular	10	4.5	
Epilepsy syndromes	28	12.7	
Toxins	8	3.6	
Unidentified	14	6.4	

## Discussion

In this study, which was conducted in a tertiary care hospital in India, the sample included 140 males and 80 females, with a male-to-female ratio of 1.75:1. This may be due to the higher male population in this region. In global studies, the male-to-female ratio is 1.35:1 [7,8]. A recent study conducted in Kerala, India, showed a male-to-female ratio of 1.05:1 [9]. Chen et al. [7] reported a male-to-female ratio of 1.18:1. All of these studies indicated male dominance in first-episode seizures.

This study found that the incidence of first-episode seizures was more common before the age of four. With an increase in age, the incidence of seizures was found to decline. This may be due to the higher susceptibility and incidence of febrile seizures as well as more central nervous system (CNS) infections and metabolic derangements among children of this age group [7]. Alakkodan [9] from India reported the highest incidence of first-episode seizures among children aged four years or younger (43.11%), followed by those aged 5-8 years (29.81%) and 9-12 years (27.06%). A study from Taiwan reported that 74.3% of children experience first-episode seizures before the age of six.

There is an increased risk of seizure in children with a family history of epilepsy or seizures. In this study, 21.8% of children had a strong positive family history of seizure or epilepsy through their first- or second-degree relatives. This finding was almost consistent with Alakkodan [9], who found a positive family history of seizures in 25.2% of cases. However, Chen et al. [7] reported a family history of seizures in only 8.2% of the cases. This may be explained by the high percentage of consanguineous marriages in India. In Inanlou et al. [10], 28.8% of children had a positive family history of seizures.

In this study, seizures were categorized based on the ILAE 2017 classification [6]. Generalized onset seizures were observed in half of the children and focal seizures in 96 (43.6%) children. Hence, generalized onset seizures are the common type of seizures among children. These findings were similar to those of other studies [7-9,11]. For instance, in Alakkodan [9], 54.12% of children presented with generalized onset seizures and 45.87% with focal onset seizures [9]. However, Chen et al. [7] reported that 75% of children had generalized onset seizures and only 5% had focal seizures. In this study, the seizure type in only six (2.7%) children was unknown. Chen et al. [7] reported that 19.7% of children had other types of seizures, which is higher than our study, as the former study included non-motor seizures in the category of other types of seizures [7].

In our study, 9% of children presented with status epilepticus as their first episode of seizure. This status epilepticus included febrile status and focal status epilepticus due to various etiologies. Alakkodan [9] reported that 27% of children had status epilepticus, which was higher than the percentage in this study. It may be explained by the fact that, in Alakkodan [9], CNS infections were found in the majority of the participants. Chen et al. [7] reported that only 0.3% of the sample (only one among 319 children) presented with status epilepticus, which contradicts other studies [7].

In this study, EEG was performed in 160 (72.72%) children who required the test. EEG was not done for some cases of febrile seizures, metabolic abnormalities, and non-cooperative children. Among these children, 24% had normal EEG results. This study showed that the majority of EEG abnormalities presented in the form of focal slowing and discharges confined to a specific brain region. Focal slowing was reported in 7.5% of children, whereas generalized non-specific slowing was observed in 23.75% of children. These results are contrary to other studies [7,9]. For example, in Alakkodan [9], 70% of children had focal discharges related to specific brain lobes, which is higher than the prevalence of focal discharges of only 33.75% in this study. This may be explained by the fact that, in Alakkodan [9], 26.6% of patients had space-occupying lesions and head injuries, while in our study, only 9% of children had space-occupying lesions. Capray et al. [12] reported that 44% of children with one or more newly diagnosed idiopathic or remote symptomatic seizures had normal initial EEG. One Indian study reported that 58.7% of children had abnormal EEG findings [13]. Chen et al. [7] reported 77.8% had normal EEG findings, which contradicts this study. In this study, epilepsy syndromes were diagnosed by EEG results along with other history and physical findings. Among the epilepsy syndromes, benign epilepsy of childhood with centrotemporal spikes with classical centrotemporal spikes was found in 10 children and Panayiotopoulos syndrome with occipital spikes in 4 children.

Half of the imaging studies presented normal reports. In this study, MRI of the brain was useful in the diagnosis of first-episode seizures in nearly 50% of the children. Neuroimaging abnormalities were found in 51.5% of children in one Indian study, whose results are similar to those of our study [14]. MRI investigation helps detect common lesions that cause seizures, such as neurocysticercosis, acute infracts focal cortical dysplasia, hemorrhage, and gliotic lesions. Arthur et al. [15] revealed only 16% of children with first episode of afebrile, unprovoked seizures showed significant MRI abnormalities. A prospective community-based cohort study revealed that only 16% of patients with epilepsy had relevant MRI abnormalities [16].

Etiological analysis was done based on the ILAE 2017 epilepsy classification framework [17]. This study showed that febrile seizures were the most common cause of first-episode seizures (41.9%), followed by pediatric epilepsy syndromes (11.8%), with the least common being immunological diseases. Hirtz et al. [11] revealed that febrile seizures were the most common cause of seizures in children, followed by trauma, epilepsy syndromes, and CNS infections. In Alakkodan [9], CNS infections (26.6%) were the most common cause, followed by febrile seizures (20.64%), seizure disorders (16%), head injuries (12.8%), and metabolic causes (8.25%). Chen et al. [7] also reported that febrile seizures (62%) were the most common cause of seizures, followed by trauma (4.3%) and epilepsy syndromes (3.7%).

In febrile seizures, 65% had their first episode of seizure between the age of one to four, which proves again that febrile seizures are quite common in this age group. This result is similar to those of Chen et al. [7] and Alakkodan [9].

Metabolic disturbances were found as the etiology of the first episode of a seizure in 6.4% of children in this study. Alakkodan [9] had a similar result for metabolic causes. In Huang et al. [18], the etiology of 11% participants for seizures was metabolic causes [18]. Chen et al. [7] reported that only 0.9% (3/319) had a metabolic etiology, which is highly contradictory to this study. However, the incidence of metabolic abnormalities such as hypoglycemia, hypocalcemia, and hyponatremia are commonly encountered in this region. Hence, metabolic evaluation should be undertaken for evaluating children who experienced seizures.

This study has several limitations. First, this was a single-center study. Second, the small sample size could not represent the entire pediatric population of India. Febrile seizures were also included in this study, and as a result, it was not possible to differentiate between febrile and afebrile seizure profiles. Considering these limitations, multicentric studies with a large study population should be conducted in the future.

## Conclusions

First-episode seizures in children cause physical, mental, and financial stress on the parents. Detailed history, examination, and appropriate investigations can help identify the etiology of seizures. It was possible to determine the etiology of the first episode of a seizure in the majority of the patients. Generalized seizures were the most common type of seizures. Febrile seizures, epilepsy syndrome, CNS infections, vascular lesions, and metabolic disorders were the main etiological factors, in that order.
